# The PP2A Catalytic Subunit PPH21 Regulates Biofilm Formation and Drug Resistance of *Candida albicans*

**DOI:** 10.3390/microorganisms13092093

**Published:** 2025-09-08

**Authors:** Jiadi Shen, Yuzhi Li, Haochen Miao

**Affiliations:** 1 Department of Endodontics, The Affiliated Stomatological Hospital of Nanjing Medical University, Nanjing 210000, China; 2 State Key Laboratory Cultivation Base of Research, Prevention and Treatment for Oral Diseases, Nanjing Medical University, Nanjing 210000, China; 3 Jiangsu Province Engineering Research Center of Stomatological Translational Medicine, Nanjing Medical University, Nanjing 210000, China; 4 Department of the Seventh Clinic, The Affiliated Stomatological Hospital of Nanjing Medical University, Nanjing 210000, China

**Keywords:** *Candida albicans*, protein phosphatases 2A, PPH21, biofilm formation, drug resistance

## Abstract

*Candida albicans* (*C. albicans*) biofilms exhibit enhanced resistance to conventional antifungal agents; however, the underlying pathogenic mechanisms warrant deeper exploration. Protein phosphatase 2A (PP2A), especially its catalytic activity, is crucial for maintaining physiological balance. This study focused on the role of the PP2A catalytic subunit coding gene *PPH21* in biofilm formation and drug resistance of *C. albicans*. The mutant strain (*pph21*Δ/Δ) was generated and identified. The oxidative stress was detected by the reactive oxygen species (ROS) and mitochondrial membrane potential (MMP). The autophagic activity was evaluated, and the autophagosomes were observed by transmission electron microscopy (TEM). The biofilm formation was measured by XTT reduction assay, crystal violet (CV) staining, and scanning electron microscopy (SEM). The susceptibility to antifungal agents was examined by XTT reduction assay and spot assay. Additionally, the antioxidant N-acetylcysteine (NAC) was applied to clarify the regulatory effect of *C. albicans* autophagy on oxidative stress. The pathogenicity of *PPH21* in oral *C. albicans* infection was evaluated through in vivo experiments. We found that *PPH21* deletion led to increased oxidative stress and autophagic activities, but it can be reversed by the application of NAC. Moreover, *PPH21* deletion also impaired the biofilm formation ability and reduced resistance to antifungal agents. Our findings revealed that *PPH21* is involved in both virulence and stress adaptation of *C. albicans*.

## 1. Introduction

*Candida albicans* (*C. albicans*), a common opportunistic pathogenic fungus, can colonize the skin, mucosal surfaces, and intestinal tract of healthy individuals [1]. However, it is also notorious for triggering persistent superficial mucosal infections or life-threatening systemic diseases, particularly in immunocompromised or immunologically deficient individuals [2]. The virulence of *C. albicans* is mainly dependent on its ability to form biofilms, which are densely packed communities of cells encased within an extracellular matrix (ECM) [3]. Alarmingly, biofilm-associated infections exhibit remarkable resistance to conventional antifungals compared to planktonic cells, posing a serious challenge to clinical antifungal therapy [4,5].

Nowadays, the commonly used antifungal agents clinically include azoles, polyenes, and echinocandins, which primarily target fungal cell membranes or cell wall biosynthesis [6]. Nevertheless, their efficacy is often compromised, largely attributed to the emergence and rise of drug-resistant strains. In recent years, despite advancements in antifungal therapeutics, the treatment of *C. albicans*-related infections remains challenging. Consequently, there is an urgent need to explore innovative therapeutic approaches or identify novel drug targets.

Autophagy is a highly conserved catabolic process widely present in eukaryotic cells, through which accumulated protein and damaged organelles are sequestered within double-membraned vesicles termed autophagosomes. These cargoes are subsequently degraded and recycled via lysosomes or vacuoles, thereby eliminating superfluous cellular components to resist stress and maintain cellular functions [7]. In *Saccharomyces cerevisiae*, autophagy can facilitate hyphal formation by alleviating endoplasmic reticulum stress, thereby modulating biofilm formation [8]. Autophagy in yeast can be induced under nutrient deficiency or stress conditions, accompanied by changes in reactive oxygen species (ROS) levels [9]. ROS are highly reactive molecules containing oxygen, including peroxides, superoxide, and hydroxyl free radicals [10]. Under normal physiological conditions, intracellular ROS is typically maintained at low levels; however, when exposed to external stimuli, accompanied by mitochondrial dysfunction, excessive ROS production is generated in cells, leading to oxidative damage [11]. Elevated ROS levels may activate autophagy to mitigate ROS-induced damage [12]. Based on this, this study speculated that oxidative stress of *C. albicans* may be regulated through autophagy to maintain cellular homeostasis.

Protein phosphatases serve as critical regulators in multiple physiological mechanisms, as well as promising therapeutic targets for various diseases. Their catalytic function entails direct dephosphorylation of protein substrates via hydrolytic cleavage of phosphate groups from amino acid residues. Protein phosphatase 2A (PP2A) is a serine–threonine phosphatase involved in the regulation of diverse cellular processes such as protein phosphorylation. The catalytic activity of PP2A is indispensable for maintaining physiological functions of the organism, which are severely compromised in the absence of or a decrease in PP2A activity [13]. In yeast, PP2A is primarily composed of three distinct subunits, including a structural subunit (PP2A-A), a regulatory subunit (PP2A-B), and a catalytic subunit (PP2A-C). Previous studies have revealed that the genome of *C. albicans* contains only one PP2A catalytic subunit (PP2A-C) encoded by *PPH21* [14], and the absence of PP2A-C usually eliminates 80–90% of PP2A activity and critically impairs cell growth [15]. Therefore, it is hypothesized that PP2A catalytic subunit PPH21 may be involved in the biofilm formation of *C. albicans*, as well as further affecting drug resistance and virulence.

This study preliminarily investigated the role of *C. albicans PPH21* in biofilm formation, drug resistance, oxidative stress responses, and autophagy by constructing the *PPH21* mutant strain (*pph21*Δ/Δ). By exploring the potential correlation between PP2A and biofilm development, this research demonstrates that PP2A-mediated regulation intersects with oxidative stress and autophagy pathways of *C. albicans*. Deletion of *PPH21* leads to decreased antioxidant capacity, which may contribute to the compromised biofilm formation and reduced antifungal resistance of *C. albicans*. Our findings identify PP2A as a critical coordinator of fungal pathogenicity and can provide novel insights and potential therapeutic targets against candidiasis.

## 2. Materials and Methods

### 2.1. Strains and Growth Conditions

The *C. albicans* strain SN152 was purchased from the American Type Culture Collection (ATCC, Manassas, VA, USA). The strain was stored as frozen stock with glycerol at −80 °C until use and routinely grown on Yeast Extract Peptone Dextrose (YPD) medium (2% peptone, 1% yeast extract, and 2% glucose) for recovery.

The *pph21*Δ/Δ mutant strain was generated from *C. albicans* SN152 using the *HIS*-*LEU*-*ARG* knockout strategy with *pSN52* and *pSN40* plasmids [16]. The plasmids were provided by the State Key Laboratory of Pharmacy Genetic Engineering, Second Military Medical University, Shanghai, China. Disruption fragments (*C. albicans HIS1* and *LEU2*) were produced by fusion PCR and transformed into *SN152* to obtain *pph21*Δ/Δ. Selective mediums (SD-HIS, SD-LEU, and SD-HIS-LEU) were employed for positive selection of the homologous recombination transformants during the construction of the mutant strain. The positive clone was confirmed by colony PCR to detect the presence of correct knockout junctions with the designed primers. Molecular characterization via DNA agarose gel electrophoresis in Supplemental Material was also performed (Figure S1). The YPD medium was prepared for *C. albicans* (SN152 and *pph21*Δ/Δ) incubation. *C. albicans* strains and plasmids used in this study are listed in Table 1.

### 2.2. Planktonic Cells Growth

The strains (*C. albicans* SN152 and *pph21*Δ/Δ) were incubated in YPD medium overnight on a shaker at 30 °C and 200 rpm to reach the logarithmic growth phase. The planktonic cells were collected, centrifuged, washed with sterile phosphate-buffered saline (PBS), and resuspended in a YPD medium. The cell suspensions were standardized to a concentration of 1.0 × 10^6^ cells/mL, and a 10 mL suspension was incubated for 24 h at 30 °C and 200 rpm. Then, *C. albicans* planktonic cells were harvested for the subsequent experiments.

### 2.3. Biofilm Formation

The biofilm was developed as previously described with slight modifications [18]. The strains were cultivated in YPD medium overnight at 30 °C and 200 rpm. The cells were harvested and resuspended in Roswell Park Memorial Institute (RPMI) 1640 medium (Gibco Ltd., Paisley, UK). The cell concentration was adjusted to 1 × 10^6^ cells/mL and inoculated into 96-well plates or culture dishes (Thermo Fisher Scientific Inc., Waltham, MA, USA). After incubation for 2 h, the plates or culture dishes were washed with sterile PBS to remove the non-adherent cells. The fresh medium was reintroduced and then the plates or dishes were incubated at 37 °C and 5% CO_2_ for 24 h to allow biofilm formation. Additionally, to clarify whether the autophagy of *C. albicans* has a regulatory effect on oxidative stress, the biofilm was also pretreated with 5 mM N-acetylcysteine (NAC, a ROS inhibitor) (Sigma-Aldrich, St. Louis, MO, USA) for 10 min [19].

### 2.4. XTT Reduction Assay

The metabolic activity of the biofilm was assessed by the 2,3-bis-(2-methoxy-4-nitro-5-sulfophenyl)-2H-tetrazolium-5-carboxanilide (XTT) reduction assay by adopting the protocol described earlier [20]. The reagent solution was prepared by mixing a 1 mg/mL XTT solution dissolved in PBS with 0.4 mM menadione solution (Sigma-Aldrich, St. Louis, MO, USA), and then added this to the wells of the 96-well plates on which the biofilm was formed. After incubation for 90 min in the dark, absorbance was measured at 490 nm using a microplate reader (SpectraMax M2, Molecular Devices, Sunnyvale, CA, USA). The optical density (OD) values refer to the metabolic activity of the biofilm, and the color change of the reagent solution is directly proportional to the number of viable cells; therefore, a greater absorbance indicates a greater population of metabolically active cells.

### 2.5. Crystal Violet (CV) Staining

Crystal violet (CV) staining of biofilm in microplate wells is one of the most widely used methods for the quantification of biofilm biomass [21], which was performed based on a previous study [22]. After biofilm formation, the 96-well plates were washed with sterile PBS three times. Subsequently, the completely dry biofilm was stained with 0.1% (*w*/*v*, in water) CV solution for 15 min at room temperature and then washed with sterile water to remove the excess CV, followed by dissolving in 33% acetic acid. The biofilm mass was quantified as a measurement of the absorbance at 570 nm using a microplate reader (SpectraMax M2, Molecular Devices, Sunnyvale, CA, USA). One well with sterile medium was used as a blank control. The assay was repeated at least three times per strain.

### 2.6. Scanning Electronic Microscopy (SEM)

The biofilm was grown on sterilized cover slips pretreated with poly-l-lysine solution measuring 8 mm in diameter and 2 mm in thickness. Then, the formed biofilm was fixed in 2.5% glutaraldehyde overnight at 4 °C. Afterwards, the cover slips were washed three times with sterile PBS and dehydrated using a graded ethanol series (30%, 50%, 70%, 80%, 90%, 95%, and 100% for 15 min, respectively). The samples were dried in air, sputter-coated with gold, and eventually visualized by scanning electron microscopy (SEM; JEOL JSM-7900F, Tokyo, Japan).

### 2.7. Antifungal Susceptibility Analysis

The susceptibility to antifungal agents was measured using an XTT reduction assay and spot assay. All the antifungal agents (fluconazole, itraconazole, amphotericin B, caspofungin, and terbinafine) were purchased from APExBIO Technology (Houston, TX, USA). The stock solutions of these agents were dissolved in dimethyl sulfoxide (DMSO) and stored at −20 °C until use. The antifungal agents used were prepared in serial 2-fold dilutions according to the M27-A4 document of the Clinical and Laboratory Standards Institute (CLSI, Berwyn, PA, USA) (2017). *C. albicans* SN152 and *pph21*Δ/Δ biofilms were allowed to develop for varying incubation times (6, 12, 24, and 48 h), prior to treatment with antifungal agents for an additional 24 h. The wells without agent treatment were set as the control group. The metabolic activity of the biofilm was also measured by XTT reduction assay to calculate the SMICs_50_ (sessile minimum inhibitory concentrations 50%), which was defined as 50% inhibition of biofilm metabolic activity compared with the control group [23].

For the spot assay, each *C. albicans* strain was prepared at a concentration of 1 × 10^7^ cells/mL, then serially diluted 10-fold, after which 3 μL of each dilution was spotted onto YPD agar plates containing various antifungal agents such as amphotericin B (6 μg/mL), fluconazole (8 μg/mL), caspofungin (0.125 μg/mL), terbinafine (16 μg/mL), and itraconazole (8 μg/mL). Growth differences of the plates were photographed after incubation for 48 h at 30 °C.

### 2.8. Reactive Oxygen Species (ROS)

The ROS level in biofilm cultured for 24 h was determined by the oxidant-sensitive agent 2′,7′-dichlorofluorescin diacetate (DCFH-DA) [24]. Non-fluorescent DCFH-DA enters cells, which is followed by gradually esterifying and oxidizing to detectable fluorescent DCF. The biofilm was incubated with DCFH-DA (Beyotime Biotechnology, Shanghai, China) for 1 h at 37 °C in the dark and then washed with PBS to remove residual dye. The fluorescence observation was obtained using a fluorescence microscope (Leica DMI3000B, Wetzlar, Germany). The fluorescence intensity of DCF (excitation, 488 nm; emission, 520 nm) was analyzed using a fluorescence spectrophotometer (SpectraMax M2, Molecular Devices, Sunnyvale, CA, USA), and then the relative ROS level was calculated.

### 2.9. Mitochondrial Membrane Potential (MMP)

MMP (ΔΨ) is one of the important pointers for indicating the structure and function of mitochondria, which can be evaluated through the JC-1 assay [25]. JC-1 (a cationic dye) serves as a reliable fluorescent probe to assess the mitochondrial ΔΨ, which changes from red fluorescent (J-aggregates, emission maximum at 590 nm) to green fluorescent (J-monomers, emission maximum at 529 nm), indicating a decrease in ΔΨ [26]. The planktonic cells and formed biofilm (SN152 and *pph21*Δ/Δ) were stained with JC-1 (Beyotime Biotechnology, Shanghai, China) at 37 °C for 30 min in the dark. Subsequently, the fluorescence images of the biofilm were observed using a fluorescence microscope (Leica DMI3000B, Wetzlar, Germany). The fluorescence intensity of planktonic cells was recorded via a flow cytometer (BD Biosciences, Franklin Lakes, NJ, USA) to determine the relative ratio of the red to green fluorescence.

### 2.10. Alkaline Phosphatase (ALP) Activity

The alkaline phosphatase (ALP) activity assay can be used as an indicator of autophagy. The ALP was activated, then detected by a microplate reader when autophagy occurred [27]. The SN152 and *pph21*Δ/Δ biofilm was collected and quantitatively measured using an ALP assay kit (Beyotime Biotechnology, Shanghai, China), which is based on the conversion of the colorless p-nitrophenyl phosphate (pNPP) substrate to the colored p-nitrophenol product after incubation with the cell samples for 30 min at 37 °C. Absorbance was recorded at 405 nm using a microplate reader (SpectraMax M2, Molecular Devices, Sunnyvale, CA, USA) to colorimetrically determine enzyme activity. The results were normalized to the total protein content and presented as nanomoles of p-nitrophenol produced per min per mg of protein (nmol/min/mg protein).

### 2.11. Transmission Electron Microscopy (TEM)

Transmission electron microscopy (TEM) was used for morphological observation of the autophagosomes in the biofilm. The biofilm was collected, washed with sterile PBS three times, and then fixed in 2.5% glutaraldehyde overnight at 4 °C, followed by post-fixing with 1% osmium tetroxide solution. Then, the samples were dehydrated in a gradient of ethanol solutions (30%, 50%, 70%, 80%, and 90% for eight min; 100% for eight min 3 times) and subsequently embedded into epoxy resins (EPON812). Ultrathin sections were cut and stained with 1% uranyl acetate for 20 min, followed by lead citrate for 5 min before the images were acquired using TEM (Carl Zeiss, Oberkochen, Germany).

### 2.12. RT-qPCR

Real-time quantitative PCR (RT-*q*PCR) was applied to analyze the expression of autophagy-related genes (ATGs) in *C. albicans* biofilm. The total RNA was extracted from the *C. albicans* biofilm using a commercial RNA extraction kit (Vazyme, Nanjing, China). Then, the integrity of the RNA of each group was assessed using a NanoDrop One spectrophotometer (Thermo Fisher Scientific Inc., Waltham, MA, USA) before reverse transcription, which ensured that the A260/A280 ratio was within the range of 1.9–2.2. Subsequent cDNA synthesis was performed with HiScript II RT SuperMix (Vazyme, Nanjing, China) following the manufacturer’s protocols. A quantitative PCR analysis of target genes and the reference gene (*β-actin*) was conducted on an ABI 7900 Fast Real-time PCR system (Applied Biosystems, Rotkreuz, Switzerland) using AceQ *q*PCR SYBR Green Mix (Vazyme, Nanjing, China). The 2^−ΔΔCt^ method was employed for relative quantification, normalizing target gene expression to the endogenous control *β-actin* [28]. All primer sequences used were synthesized by Shanghai General BioTech Co., Ltd. (Shanghai, China) (Table 2).

### 2.13. Western Blotting

Protein extracts from the *C. albicans* biofilm were isolated via immunoprecipitation for the Western blot analysis [29]. Protein concentrations were quantified with a BCA assay kit (Beyotime Biotechnology, Shanghai, China). Protein samples were mixed with a one-fifth volume of 5 × sample buffer (Beyotime Biotechnology, Shanghai, China) for SDS-PAGE. The samples were boiled for 3–5 min and separated by SDS-PAGE on 10% acrylamide gels. Subsequently, protein samples were electro-transferred to polyvinylidene fluoride (PVDF) membranes (Millipore, Billerica, MA, USA). Following 1 h of blocking at room temperature with 5% skimmed milk in TBST (2.41% Tris, 8% NaCl, 1% Tween-20), membranes were incubated overnight at 4 °C with primary antibodies (ATG1, ATG13, ATG17, ATG27; 1:500 dilution, Dia-An Biotech, Wuhan, China), followed by 1 h of incubation with HRP-conjugated secondary antibodies (1:10,000 dilution). Protein bands were visualized via a chemiluminescence system (Merck & Co., Inc., Kenilworth, NJ, USA), with GAPDH (Bioworld, Minneapolis, MN, USA) serving as the reference protein. 

### 2.14. Construction of Murine Oral Candidiasis Model

All animal experiments were approved by the Institutional Animal Care and Use Committee of Nanjing Medical University (IACUC-2307018). Six-week-old female ICR mice were acquired from the Medical Laboratory Animal Center of Nanjing Medical University. The mice were raised in cages containing 5 to 6 animals and were free to get food and water. The photo-periods were adjusted to a 12 h light and 12 h darkness daily cycle, and the environmental temperature was maintained at 21 °C.

The construction of the murine oral candidiasis model was carried out based on the protocol used by Takakura et al. [30]. In total, sixty mice were randomly divided into three groups (normal, SN152 and *pph21*Δ/Δ), with 20 mice in each group (5 at each time-point: days 1, 3, 5, and 7), and the sample size was decided based on a previous study [31]. To enhance murine susceptibility to *C. albicans*, immunosuppression was induced by administering 10 g/L of prednisolone (Aladdin, Shanghai, China) 24 h before inoculation. Tetracycline hydrochloride (0.83 g/L; Aladdin, Shanghai, China) was supplemented in drinking water throughout the experimental period. Prior to infection, mice were anesthetized by intramuscular injection of 50 μL chlorpromazine (2 g/L; Aladdin, Shanghai, China). Oral candidiasis was established by swabbing all mucosal surfaces (including buccal, lingual, and palatal) with cotton applicators saturated in a *C. albicans* suspension (2 × 10^8^ CFU/mL). The murine oral candidiasis model was considered successfully established upon pseudomembrane lesion formation in the oral cavity, allowing for subsequent experiments; mice failing this criterion were euthanized and excluded. The uninfected group (normal) received identical handling procedures as the infected groups (SN152 and *pph21*Δ/Δ), with sterile 0.9% NaCl substituted for the *C. albicans* suspension during oral inoculation.

A macroscopic assessment of murine tongue lesions employed the pseudomembrane scoring system from 0 to 4, as described in a previous study [30]. The progression of the infection in the murine oral cavities was quantified via viable *C. albicans* cells using a microbiological evaluation, calculated as a colony-forming unit (CFU) value [30]. Additionally, excised tongues underwent 4% paraformaldehyde fixation and paraffin embedding. Serial 5 μm sections were prepared, stained with Periodic Acid-Schiff (PAS), and examined histopathologically under a light microscope (Leica DM4000, Wetzlar, Germany).

### 2.15. Statistical Analysis

The quantitative assessment tests were conducted thrice, and the results, presented as the means ± standard deviations (SDs), were compared. A one-way analysis of variance (ANOVA) was employed to compare the statistical significance of differences among groups. Differences were considered statistically significant (*p*-value < 0.05), which were analyzed using SPSS 22.0 software (SPSS Inc., Chicago, IL, USA). The corresponding graphs were constructed using GraphPad Prism 5.0.

## 3. Results

### 3.1. PPH21 Deletion Led to Increased Oxidative Stress in C. albicans Biofilm

**Figure 1:**
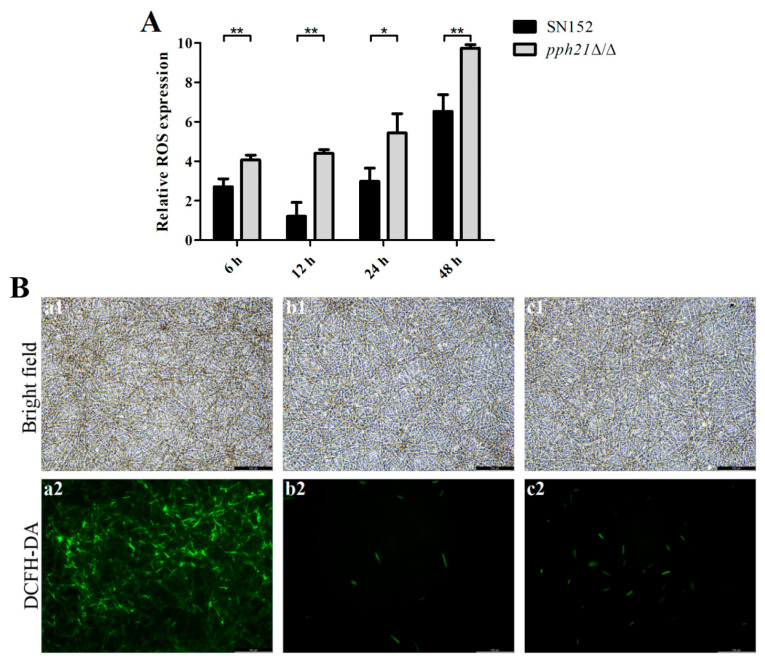
The ROS levels of SN152 and *pph21*Δ/Δ. (**A**) The relative ROS expression at different time phases (6 h, 12 h, 24 h, and 48 h), * *p* < 0.05; ** *p* < 0.01. (**B**) Representative DCFH-DA fluorescence images of 24 h biofilm: (**a1**,**a2**) SN152 Rosup; (**b1**,**b2**) SN152; (**c1**,**c2**) *pph21*Δ/Δ; 1: bright field; 2: DCFH-DA. Scale bar = 100 µm. The ROS level in *pph21*Δ/Δ biofilm was significantly higher than that in the control (SN152), accompanied by the increased green fluorescence intensity observed.

The generation of ROS was examined by the changes in the fluorescence, resulting from the oxidation of DCFH-DA dye into DCF. As depicted in Figure 1A, the *pph21*Δ/Δ mutant strain displayed considerable upsurges in the intracellular ROS level at different time periods (6 h, 12 h, and 48 h: *p* < 0.01; 24 h: *p* < 0.05), as compared to the control (SN152). Additionally, the increased oxidative stress of *pph21*Δ/Δ was also visually confirmed by intensified green fluorescence, manifested as the green fluorescence intensity in the 24 h biofilm of *pph21*Δ/Δ being stronger than that of SN152 (Figure 1B).

**Figure 2:**
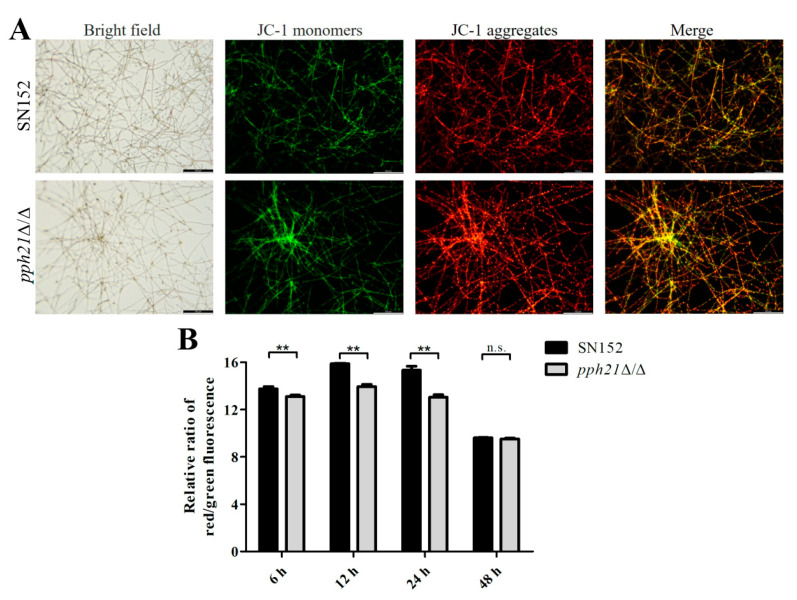
Changes of ΔΨ in SN152 and *pph21*Δ/Δ biofilms: (**A**) representative JC-1 fluorescence images of 24 h biofilm, scale bar = 100 µm; (**B**) relative ratio values of JC-1 red to green fluorescence at different time phases (6 h, 12 h, 24 h, and 48 h). The increased green fluorescence intensity was detected in *pph21*Δ/Δ rather than in the control (SN152), along with the significantly decreased relative red/green fluorescence ratios at 6, 12, and 24 h (*p* < 0.01); ** *p* < 0.01; n.s.: no significant difference.

The JC-1 staining results also showed that *pph21*Δ/Δ presented a stronger green fluorescence intensity than SN152 (Figure 2A), consistent with the ROS level results (Figure 1). In addition, the relative red/green fluorescence ratio values of *pph21*Δ/Δ decreased significantly at 6, 12, and 24 h (*p* < 0.01) (Figure 2B). The findings above indicate that the deletion of *PPH21* resulted in the ROS accumulation in the *C. albicans* biofilm, accompanied by a decrease in ΔΨ.

### 3.2. PPH21 Deletion Induced Increased Autophagic Activity in C. albicans Biofilm

**Figure 3:**
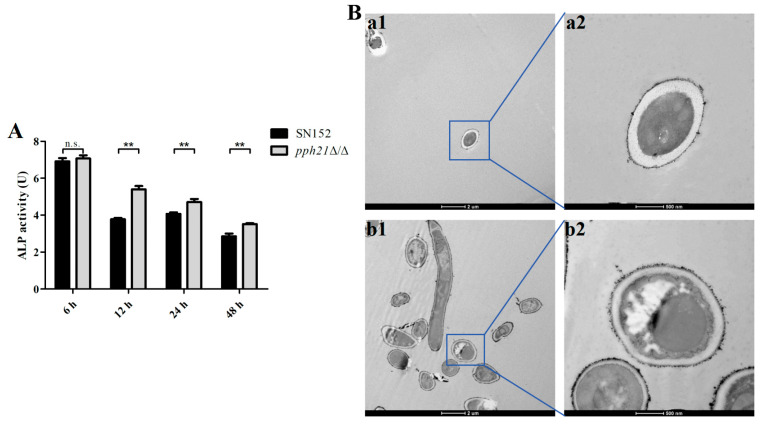
The autophagic activities of SN152 and *pph21*Δ/Δ biofilms. (**A**) ALP activity levels at different time phases (6 h, 12 h, 24 h, and 48 h). The *pph21*Δ/Δ biofilm exhibited significantly higher ALP activity compared to SN152 at 12, 24, and 48 h (*p* < 0.01); ** *p* < 0.01; n.s.: no significant difference. (**B**) Typical TEM images of autophagosomes in 24 h biofilm: (**a1**,**a2**) SN152; (**b1**,**b2**) *pph21*Δ/Δ; 1: scale bar = 2 µm; 2: scale bar = 500 nm.

**Figure 4:**
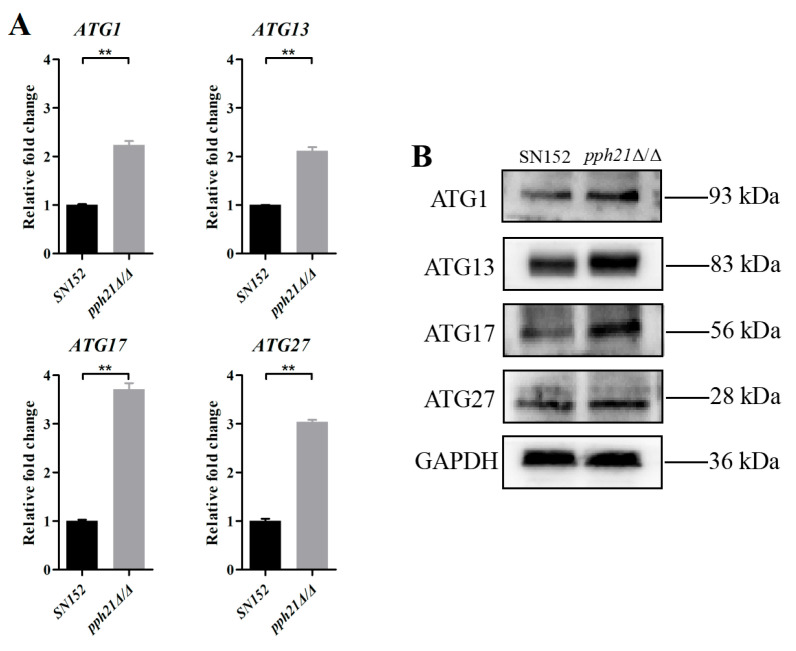
The expression levels of ATG genes and proteins of SN152 and *pph21*Δ/Δ biofilm. (**A**) Comparison of ATG gene expression levels between SN152 and *pph21*Δ/Δ via RT-*q*PCR; ** *p* < 0.01. (**B**) Comparison of ATG protein levels between SN152 and *pph21*Δ/Δ via Western blotting. The expression levels of *ATG1*, *ATG13*, *ATG17*, and *ATG27* in *pph21*Δ/Δ biofilm were significantly upregulated (*p* < 0.01), and the protein levels also increased.

To assess whether the deletion of *PPH21* affects autophagic activity levels, we measured the ALP activity, along with the autophagosomes in the biofilms (Figure 3). The ALP activity of the *pph21*Δ/Δ biofilm was significantly higher than that of the SN152 biofilm (at 12, 24, and 48 h) (*p* < 0.01; Figure 3A). TEM can be utilized to detect autophagosomes and examine alterations in their ultra-structural morphology when autophagy occurs. TEM showed that autophagosome formation was observed in *pph21*Δ/Δ, although their membranes and boundaries appeared indistinct, while there were fewer and no obvious autophagosomes in SN152 (Figure 3B). Moreover, relative to SN152, the *pph21*Δ/Δ biofilm presented a significant increase in the expression levels of *ATG1*, *ATG13*, *ATG17*, and *ATG27* (*p* < 0.01; Figure 4A), concomitant with elevated protein levels of ATG1, ATG13, ATG17, and ATG27 (Figure 4B).

Compared to the control (SN152), *pph21*Δ/Δ exhibited increased autophagic activities. Given the concomitant increase in oxidative stress observed in *pph21*Δ/Δ, our findings suggested a potential regulatory interplay may exist between oxidative stress and autophagy in *C. albicans*.

### 3.3. The Increased Oxidative Stress and Autophagic Activity of pph21Δ/Δ Biofilm Was Reversed by ROS Inhibitor (NAC)

**Figure 5:**
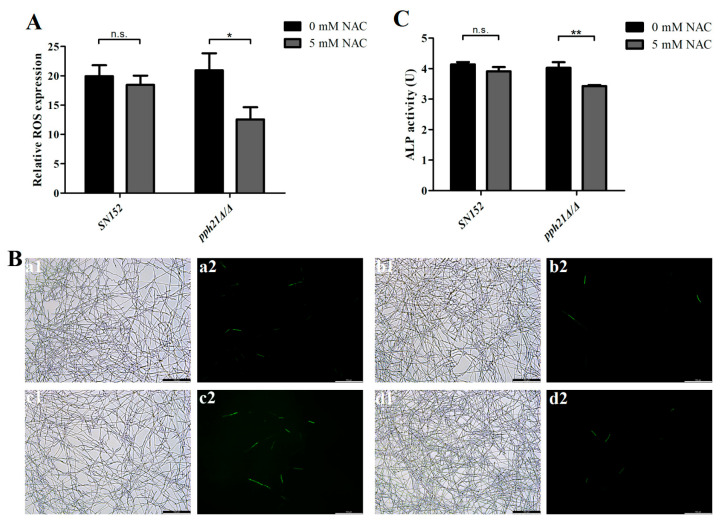
The ROS level and autophagic activity of SN152 and *pph21*Δ/Δ biofilms after pretreatment with NAC. (**A**) The relative ROS expression of the 24 h biofilm; * *p* < 0.05; n.s.: no significant difference. (**B**) Representative DCFH-DA fluorescence images of the 24 h biofilm: (**a1**,**a2**) SN152; (**b1**,**b2**) SN152 + NAC; (**c1**,**c2**) *pph21*Δ/Δ; (**d1**,**d2**) *pph21*Δ/Δ + NAC; 1: bright field; 2: DCFH-DA. Scale bar = 100 µm. (**C**) The ALP activity of the 24 h biofilm. The ROS level and ALP activity levels of the *pph21*Δ/Δ + NAC biofilm were lower than those of the untreated group (*pph21*Δ/Δ); ** *p* < 0.01; n.s.: no significant difference.

The ROS inhibitor (NAC) treatment significantly reduced the intracellular ROS level in *pph21*Δ/Δ (*p* < 0.05; Figure 5A), accompanied by decreased green fluorescence intensity (Figure 5B) and ALP activity (Figure 5C). In contrast, the control strain (SN152) presented no statistically significant reductions in either ROS level or ALP activity following NAC exposure (*p* > 0.05). The above results indicated that the elevated oxidative stress and autophagic activity observed in *pph21*Δ/Δ may be attenuated by ROS inhibition (NAC), further supporting a potential bidirectional regulation of these two pathways in *C. albicans*.

### 3.4. PPH21 Deletion Reduced the Biofilm Formation Ability of C. albicans

**Figure 6:**
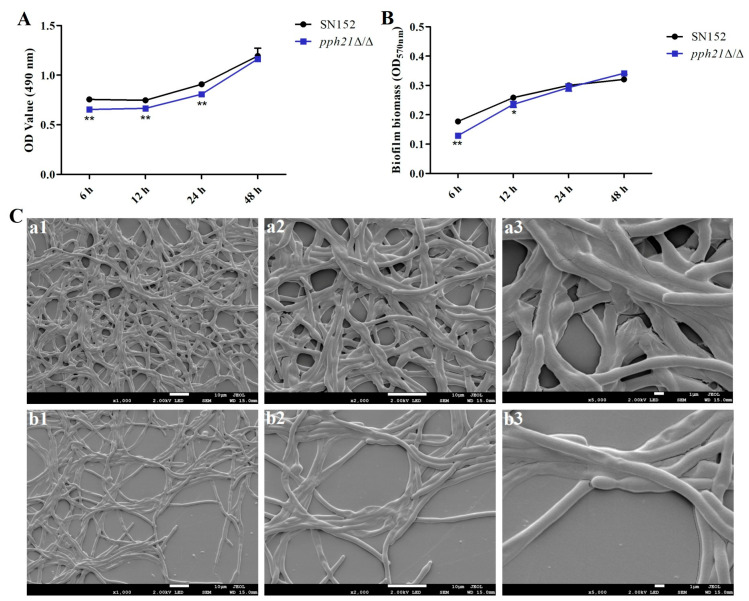
The biofilm formation ability of SN152 and *pph21*Δ/Δ biofilms. Comparison of biofilm formation between SN152 and *pph21*Δ/Δ biofilms by XTT reduction assay (**A**) and crystal violet (CV) staining (**B**); * *p* < 0.05, ** *p* < 0.01: compared with SN152. (**C**) The morphological changes of SN152 and *pph21*Δ/Δ biofilms detected with SEM, whereby the density of hyphae in the *pph21*Δ/Δ biofilm was lower than that of the control (SN152); (**a1**–**a3**): SN152; (**b1**–**b3**): *pph21*Δ/Δ; magnification: 1: 1000×; 2: 2000×; 3: 5000×.

The *pph21*Δ/Δ mutant strain displayed impaired biofilm formation ability relative to the control strain (SN152). A quantitative assessment via XTT reduction assay revealed that the *pph21*Δ/Δ biofilm exhibited significantly reduced OD values at 6 h, 12 h, and 24 h (*p* < 0.01) compared to SN152, whereas no significant variation was observed at 48 h (*p* > 0.05) (Figure 6A). Additionally, the CV staining results showed significantly lower OD values in the *pph21*Δ/Δ biofilm at 6 h (*p* < 0.01) and 12 h (*p* < 0.05), with no statistical differences at 24 h and 48 h (*p* > 0.05) (Figure 6B).

Additionally, SEM further demonstrated attenuated hyphal development and decreased mycelial density in the *pph21*Δ/Δ biofilm (Figure 6C), consistent with the aforementioned XTT findings. The compromised *pph21*Δ/Δ biofilm architecture suggested that *PPH21* may play a crucial role in biofilm formation and maintaining the structural integrity of *C. albicans*.

### 3.5. PPH21 Deletion Showed Increased Susceptibility to Antifungal Agents

We also investigated whether the deletion of *PPH21* altered the susceptibility of *C. albicans* to antifungal agents. The XTT assay results showed that compared with SN152, *pph21*Δ/Δ showed lower SMIC_50_ values against fluconazole (6 h, 12 h, and 24 h), itraconazole (6 h, 24 h, and 48 h), amphotericin B (48 h), and caspofungin and terbinafine (12 h and 24 h), indicative of reduced antifungal resistance. Moreover, both SN152 and *pph21*Δ/Δ exhibited a time-dependent elevation in SMIC_50_ values against antifungal agents (Table 3).

**Figure 7:**
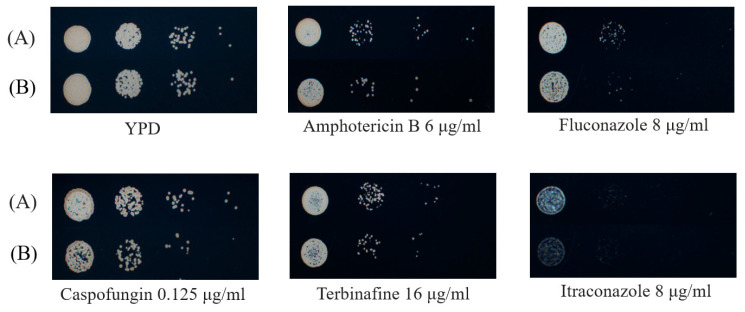
The susceptibility of SN152 and *pph21*Δ/Δ to the antifungal agents (amphotericin B, fluconazole, caspofungin, terbinafine, and itraconazole) using the spot assay. The mutant strain (*pph21*Δ/Δ) failed to survive in the presence of these agents mentioned above, as compared to the control strain (SN152): (**A**) SN152; (**B**) *pph21*Δ/Δ.

In addition, the spot assay analysis revealed that *pph21*Δ/Δ, as well as SN152, showed comparable growth on YPD agar plates; however, when exposed to antifungal agents (amphotericin B, fluconazole, caspofungin, terbinafine, and itraconazole), *pph21*Δ/Δ demonstrated reduced viability compared to SN152 (Figure 7). These findings indicate that *PPH21* deletion enhanced the susceptibility to antifungal agents, ultimately resulting in the mutant strain’s failure (*pph21*Δ/Δ) to survive in the presence of antifungals.

### 3.6. PPH21 Deletion Presented Weakened Pathogenicity of C. albicans and Reduced the Oral Infection of Murine Tongues

**Figure 8:**
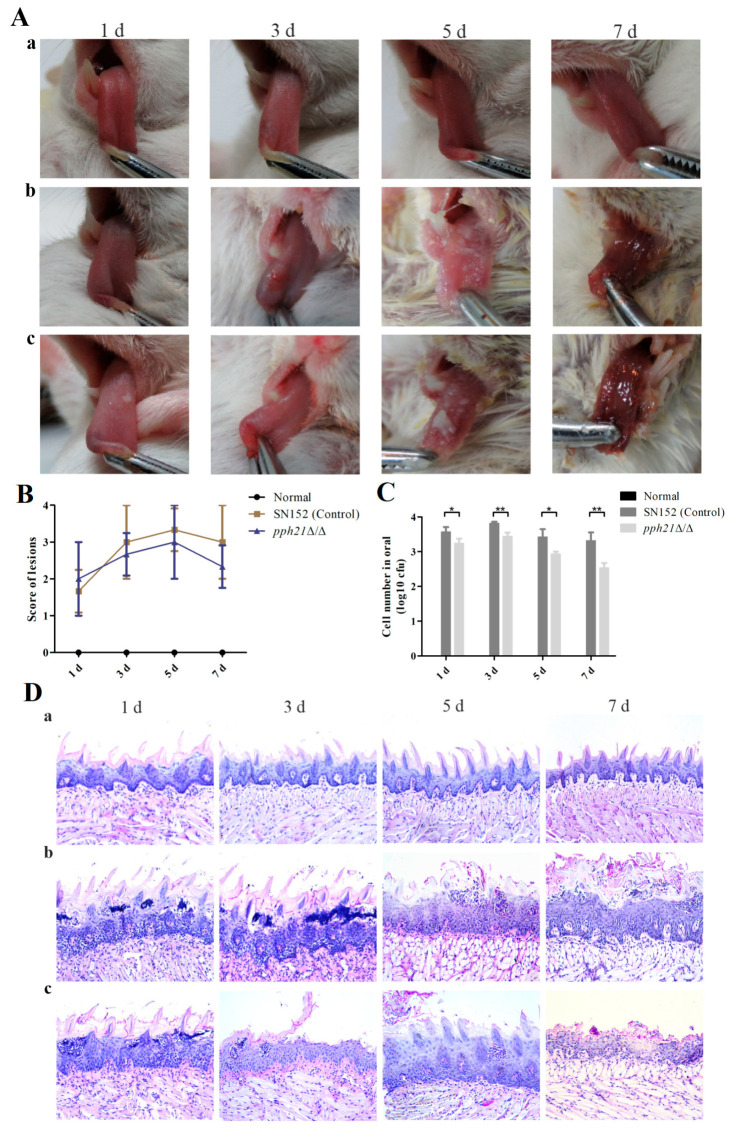
The pathogenicity of SN152 and *pph21*Δ/Δ to murine models. The *pph21*Δ/Δ mutant strain showed reduced virulence in a murine model of oral candidiasis. (**A**) Macroscopic observation of white patches of the lesions on murine tongues after *C. albicans* oral infection; (**a**) normal; (**b**) SN152; (**c**) *pph21*Δ/Δ. (**B**) Changes in the lesion score on the tongues. (**C**) The CFUs of *C. albicans* isolated from the murine oral cavity. No viable *C. albicans* cells (0 CFU) were detected in the uninfected group (normal) (no black bar shown in Figure 8C); * *p* < 0.05; ** *p* < 0.01. (**D**) Histopathological observation of a longitudinal section of a murine tongue (×200 magnification). The tissue sections were stained with PAS; (**a**) normal; (**b**) SN152; (**c**) *pph21*Δ/Δ.

The pathogenicity of the *pph21*Δ/Δ mutant strain was further assessed through in vivo experiments. Compared with the normal group, both infected groups (SN152 and *pph21*Δ/Δ) presented with pseudomembrane lesions on the dorsum of murine tongues beginning at day 3 post-infection (Figure 8A). The *pph21*Δ/Δ group exhibited lower scores for tongue lesions than SN152 at days 3, 5, and 7 (Figure 8B). The numbers of CFUs in oral cavities of the infected groups (SN152 and *pph21*Δ/Δ) peaked at day 3, followed by progressive declines at days 5 and 7. Notably, *pph21*Δ/Δ mice demonstrated substantially reduced CFUs relative to SN152 across all time-points (days 1, 3, 5, and 7; Figure 8C). The histopathological analysis revealed that more hyphae of *C. albicans* on the tongue surfaces were observed in SN152 than in *pph21*Δ/Δ, along with concomitant epithelial disruption, whereas *pph21*Δ/Δ showed diminished fungal invasion and the normal groups maintained intact architecture without hyphal presence (Figure 8D).

## 4. Discussion

The high morbidity and mortality caused by *C. albicans* represent a critical challenge in clinical fungal infections that urgently requires resolution. Biofilms, the predominant pathogenic forms of *C. albicans*, demonstrate elevated drug resistance and reduced susceptibility to conventional antifungal agents compared to the planktonic cells. Therefore, elucidating the potential mechanisms underlying biofilm-associated drug resistance in *C. albicans* is of significant importance for advancing strategies to treat and prevent clinical infections caused by this pathogen.

While autophagy has been extensively studied in mammals, its molecular regulatory mechanisms were initially identified in yeast [32]; currently, more than 30 autophagy-related genes (ATG) have been characterized [33]. Autophagy is essential for surviving nutrient deficiency or starvation to maintain cellular viability. Previous studies have revealed that multiple ATGs are significantly upregulated in *C. albicans* biofilms compared to the planktonic cells, as determined by transcriptome sequencing analyses, suggesting a potential correlation between autophagy and biofilm formation in *C. albicans* [34]. However, the role of autophagy in biofilm development and drug resistance, as well as the underlying molecular regulatory mechanisms, remains inconclusive and warrants further elucidation.

ROS play an important role in cellular processes such as differentiation and development, commonly serving as an indicator of intracellular stress levels [35]. Mitochondria, the primary organelles responsible for ROS generation, are a major contributor to oxidative damage in cells, making mitochondrial dysfunction closely associated with ROS [36]. In this study, deletion of *PPH21* induced ROS accumulation accompanied by a decrease in MMP, indicating that *PPH21* modulates oxidative stress homeostasis in *C. albicans*. The absence of *PPH21* weakens its antioxidant defense capacity, ultimately leading to elevated oxidative stress levels.

Fortunately, excessive ROS accumulation can trigger autophagy to eliminate cellular ROS overload, thereby mitigating oxidative damage [37]. In *pph21*Δ/Δ, the activation of the autophagy–ROS axis may serve as a conserved stress response mechanism, essential for survival under diverse stress conditions (e.g., nutrient deprivation, ER stress). Consistently, *pph21*Δ/Δ exhibited increased autophagic activity levels, indicating that *PPH21* deletion can induce ROS-dependent autophagy as an adaptive survival strategy, which subsequently enhances oxidative stress resistance. To further validate this regulatory effect, NAC was employed, a potent ROS scavenger that can effectively suppress ROS production [19]. Notably, the autophagic activities in *pph21*Δ/Δ decreased concomitantly with ROS reductions upon NAC treatment, suggesting that NAC can reverse the elevated oxidative stress and autophagy of *pph21*Δ/Δ. These findings further provide compelling evidence for the autophagy–ROS regulatory axis in *C. albicans*, which dynamically coordinates cellular responses to maintain survival under stress conditions. However, further investigations should resolve the molecular mechanisms between autophagy and oxidative stress responses in *C. albicans* and whether the *PPH21*-related autophagy–ROS regulatory axis contributes to clinical antifungal persistence.

Additionally, *pph21*Δ/Δ exhibited diminished biofilm formation capacity, along with enhanced susceptibility to antifungal agents (fluconazole, itraconazole, terbinafine, amphotericin B, and caspofungin), which may be attributed to increased oxidative stress in *pph21*Δ/Δ compared to the control strain (SN152). Nevertheless, *C. albicans* (both SN152 and *pph21*Δ/Δ) still presented progressive enhancement of resistance during biofilm maturation. When exposed to external stimuli (e.g., drug treatment), the intracellular oxidative stress increases, and immoderate ROS generation can induce oxidative damage to mitochondria [38]. Excessive intracellular accumulation of ROS triggers lipid peroxidation [39], resulting in damage to cell membranes that ultimately impairs normal cellular metabolism and physiological functions [40], consequently inhibiting fungal growth and reducing drug resistance. Furthermore, a diminished cell density may directly disrupt biofilm development [41]. Concurrently, compromised membrane integrity facilitates intracellular drug penetration, enhances drug influx [42], and eventually reduces antifungal tolerance in *C. albicans*.

Furthermore, a murine oral candidiasis model was established to investigate the pathogenicity of *PPH21* in *C. albicans* infections. Both infected groups (SN152 and *pph21*Δ/Δ) developed pseudomembrane lesions on the tongue surface from day 3 post-infection, coinciding with peak fungal burdens in oral cavities. However, on days 5 and 7, the progression of infection was attenuated, as evidenced by a decrease in oral CFU, presumably attributable to progressive host immune reconstitution in the later stage of infection, leading to a certain resistance against *C. albicans* [43]. Compared to SN152, the *pph21*Δ/Δ mutant strain displayed reduced virulence, resulting in diminished infection severity in murine. However, further studies are needed to clarify the potential impact of *C. albicans PPH21* on systemic infections.

## 5. Conclusions

This study constructed the *pph21*Δ/Δ mutant strain in *C. albicans* to further elucidate the role of *PPH21* in biofilm formation, drug resistance, oxidative stress response, and autophagy regulation. Our findings revealed the importance of the autophagy–ROS regulatory axis in *C. albicans*, where *PPH21* deficiency-induced ROS accumulation activates autophagy, which in turn mitigates ROS-mediated cellular damage to maintain homeostasis. Moreover, *PPH21* deletion impaired biofilm formation and reduced resistance to antifungal agents, uncovering its critical involvement in both the virulence and stress adaptation of *C. albicans*, expected to be a potential therapeutic target for *C. albicans*-related infections.

## Figures and Tables

**Table 1:** *C. albicans* strains and plasmids used in this study.

	Genotype	Reference
*C. albicans* strains		
SN152	*arg4/arg4 leu2/leu2 his1/his1* *URA3/ura3::imm434* *IRO1/iro1::imm434*	[17]
*pph21*Δ/Δ	*arg4/arg4 leu2/leu2 his1/his1* *URA3/ura3::imm434* *IRO1/iro1::imm434* *PPH21::C.d.LEU2/pph21::C.d.HIS1*	this study
Plasmids		
*pSN52*	With *HIS1* marker	[17]
*pSN40*	With *LEU2* marker	[17]

**Table 2:** Primers and sequence used in the present study.

Primer Name	Sequence (5′→3′)
*ATG1*-F	TACAACCCAACTGAGCGGAT
*ATG1*-R	GTAGTGGGTGATGGGCTTCT
*ATG13*-F	GCCAAGACTACGGGGTATGA
*ATG13*-R	AAGCATTGGAATTGCGTCGA
*ATG17*-F	TTCAACGCCTTCCAGCAA
*ATG17*-R	TGGTTTGATCTCTGGCATTGA
*ATG27*-F	ACTCCAACAGCTATCTCGCA
*ATG27*-R	TATAACGTCGCCAACCCT
*β-actin*-F	GACCAAGAAGACATCAAGGTATCAT
*β-actin*-R	GTGTTCAATTGGGTATCTCAAG

**Table 3:** The susceptibilities of SN152 and *pph21*Δ/Δ biofilms to antifungal agents (SMIC50 (μg/mL)).

Strains	SMIC_50_ of Antifungal Agents (µg/mL)			
	6 h	12 h	24 h	48 h
**fluconazole**				
SN152	512	1024	1024	1024
*pph21*Δ/Δ	256	512	512	1024
**itraconazole**				
SN152	512	512	1024	1024
*pph21*Δ/Δ	256	512	512	512
**amphotericin B**				
SN152	2	2	4	8
*pph21*Δ/Δ	2	2	4	4
**caspofungin**				
SN152	16	32	64	64
*pph21*Δ/Δ	16	16	32	64
**terbinafine**				
SN152	256	512	1024	1024
*pph21*Δ/Δ	256	256	512	1024

## Data Availability

The original contributions presented in this study are included in the article/Supplementary Materials. Further inquiries can be directed to the corresponding author.

## Supplementary Materials

The following supporting information can be downloaded at: https://www.mdpi.com/article/10.3390/microorganisms13092093/s1, Figure S1: Molecular characterization of the *pph21*Δ/Δ mutant strain.
