# Supplementary material for: The PP2A Catalytic Subunit PPH21 Regulates Biofilm Formation and Drug Resistance of Candida albicans
Source: Microorganisms. 2025 Sep 8;13(9):2093. doi: 10.3390/microorganisms13092093 (PMC12473098; doi:10.3390/microorganisms13092093)
Supplement: Supplementary file 1 [file microorganisms-13-02093-s001.zip › microorganisms-3786455-supplementary.pdf]

Supplemental Materials

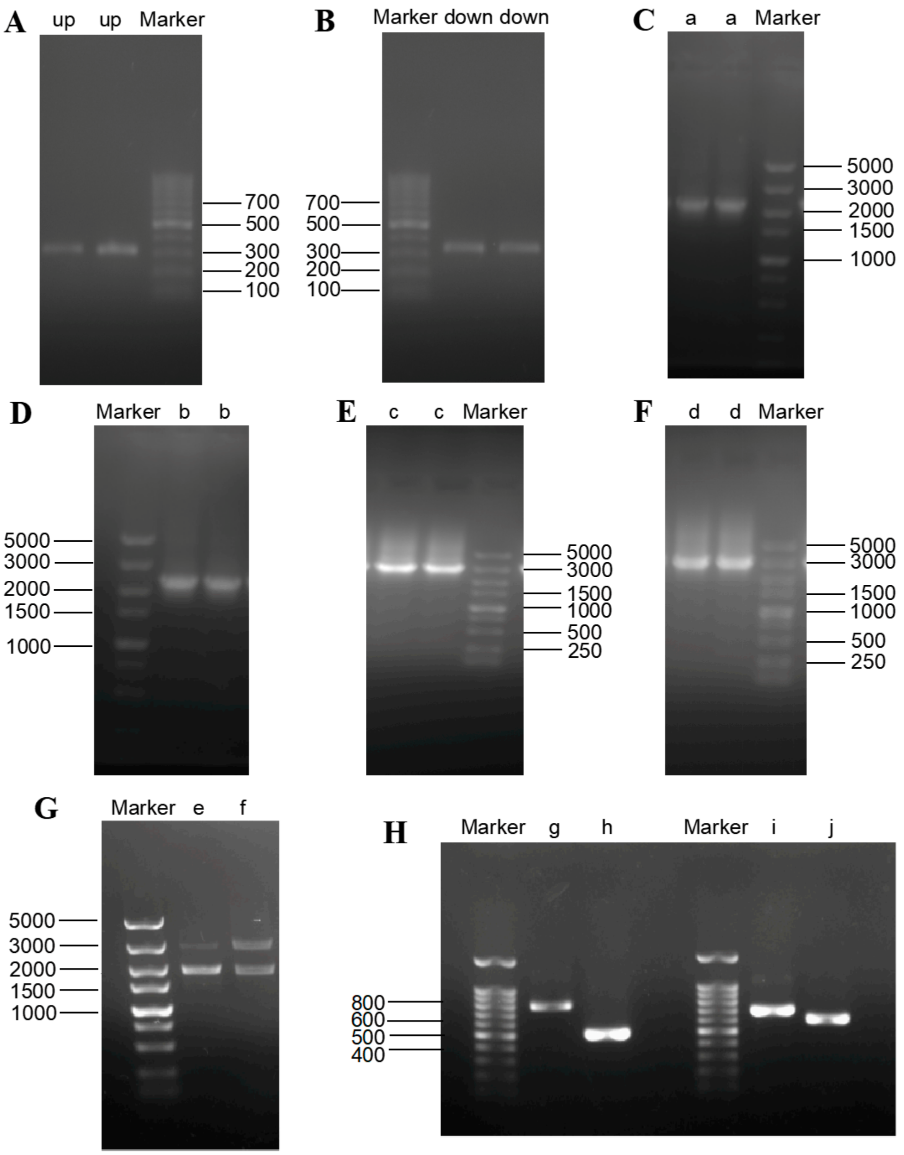

Figure S1. Molecular characterization of the *pph21*Δ/Δ mutant strain. (**A**,**B**) Identification of upstream and downstream homology arm fragments of *PPH21* (301 bp and 312 bp); (**C**,**D**) Identification of *HIS1* (2388 bp, lane a) and *LEU2* (2307 bp, lane b) fragments; (**E**) Identification of *PPH21* fusion PCR product (HIS) (3001 bp, lane c); (**F**) Identification of *PPH21* fusion PCR product (LEU) (2920 bp, lane d); (**G**) Identification of *PPH21* single-arm knockout strain (*pph21*Δ): SN152 has a bright band at 1922 bp (lane e), and *pph21*Δ has two bright bands at 1922 bp and 3001 bp (lane f); (**H**) Identification of UP-HIS-DOWN upstream and downstream connection: HIS upstream fragment (785 bp, lane g), HIS downstream fragment (494 bp, lane h); Identification of UP-LEU-DOWN upstream and downstream connection: LEU upstream fragment (696 bp, lane i), LEU downstream fragment (597 bp, lane j).
